# A machine learning algorithm to predict the success of a second microsurgical testicular sperm extraction

**DOI:** 10.1097/MS9.0000000000003560

**Published:** 2025-07-10

**Authors:** Akef Obeidat, Belal Nedal Sabbah, Hammam Mandourah, Mohammad Alghafees, Ahmad Nedal Sabbah, Amro Hajja, Abdurrahman Ouban, Wael Alkattan, Mohammed Ali Omar, Hytham Mubarak Abdalla, Abdalrahman Abuzubida, Safwan Urooj Abbasi, Zeyad Alkhneizan, Fahad Alhussain, Faisal Alsaleh, Said Kattan, Naif Alhathal

**Affiliations:** a Alfaisal University, College of Medicine, Riyadh, Saudi Arabia; b Urology, King Abdulaziz University Hospital, Jeddah, Saudi Arabia; c Urology, King Abdulaziz Medical City, Riyadh, Saudi Arabia; d Urology, King Faisal Specialist Hospital and Research Centre, Riyadh, Saudi Arabia; e College of Medicine, King Saud University Medical City, Riyadh, Saudi Arabia

## Abstract

**Introduction:**

Testicular sperm extraction (TESE) is a common procedure for retrieving sperm in men with azoospermia. However, the success rates of a second TESE following an initial unsuccessful attempt remain low. This study aims to develop and evaluate a machine learning algorithm to predict the success of a second microsurgical TESE (microTESE).

**Methods:**

Medical records of 47 patients who underwent a second microTESE were analyzed. The dataset included variables such as procedure side, histopathology, preoperative Follicle-stimulating hormone (FSH) and testosterone levels, testicular volume, and comorbidities. Supervised machine learning algorithms, including support vector machine (SVM), were employed to predict the success of the second microTESE. The dataset was split into training (80%) and testing (20%) sets.

**Results:**

The SVM model achieved an accuracy of 80% after hyperparameter tuning. Bilateral procedures and longer intervals between surgeries were associated with higher success rates, while a history of cancer correlated with negative outcomes. FSH and testosterone levels were also identified as predictive factors. The SVM model’s feature importance analysis highlighted histopathology, varicocele, hormone levels, and the interval between procedures as highly correlated with the success of a second microTESE.

**Discussion:**

The machine learning model accurately predicted the presence or absence of spermatozoa in patients with non-obstructive azoospermia undergoing a second microTESE. The findings are consistent with previous studies and provide valuable insights into the predictive factors for the success of a second microTESE. However, the study’s limitations include selection bias and reliance on retrospective data.

**Conclusion:**

The SVM model shows promise in predicting the success of a second microTESE by incorporating factors such as age, hormonal levels, testicular volume, and genetic evaluation. Further validation and refinement are needed to ensure the model’s accuracy and applicability across different populations.

## Introduction

Azoospermia, defined as the absence of spermatozoa in the sediment of a centrifuged ejaculation sample, is a significant cause of male infertility^[1]^. Testicular sperm extraction (TESE) is commonly recommended to retrieve fully developed germ cells for use in intracytoplasmic sperm injection^[2,3]^. Among the surgical techniques available, conventional TESE (cTESE) and microsurgical TESE (microTESE) are prominent, with microTESE utilizing a surgical microscope to identify seminiferous tubules likely to contain complete spermatogenesis^[4]^. However, both methods are invasive and carry risks such as hematoma, infection, vascular injury, and testosterone deficiency^[4]^. Consequently, TESE should only be pursued following a comprehensive evaluation of the couple’s infertility, thorough patient education, and multidisciplinary consultation. Current success rates for extracting spermatozoa from testicular tissue using these techniques are approximately 50%^[5,6]^.

**HIGHLIGHTS**

- Developed a support vector machine model with 80% accuracy to predict the success of a second microsurgical testicular sperm extraction in azoospermic men.
- Identified bilateral procedures, longer intervals between surgeries, and no history of cancer as positive predictors.
- Highlighted the importance of Follicle-stimulating hormone and testosterone levels as predictive factors for successful sperm retrieval.

Despite these efforts, sperm retrieval through rescue micro-TESE following an unsuccessful cTESE has been reported with a success rate of 45.7%^[7]^. Meta-analyses have shown that microTESE is 1.5 times more likely to detect sperm compared to cTESE^[4]^. Although microTESE has shown superior sperm recovery rates, data on repeat microTESE following an initial unsuccessful procedure are limited, with few studies involving small patient cohorts^[8–11]^. Preoperative evaluations typically consider clinical and hormonal factors, yet variables such as age, body mass index (BMI), total testosterone, and prolactin levels have not been reliably indicative of spermatozoa presence in testicular tissue^[12,13]^. In contrast, diminished testicular volume, elevated Follicle-stimulating hormone (FSH) levels, and reduced inhibin B levels have been associated with lower likelihoods of successful TESE^[14,15]^. Additionally, abnormal karyotypes and microdeletions in the azoospermia factor region are found in 6%–18% of azoospermic individuals^[16,17]^. The roles of cryptorchidism history and smoking habits remain inconclusive^[18,19]^.

To date, no single factor has demonstrated reliable predictive capability for TESE success, highlighting the need for better predictive tools^[20–23]^. Given the financial burden associated with the procedure, there is a critical need for improved predictive models. Advances in computing power and machine learning (ML) techniques offer new opportunities to enhance predictive accuracy by integrating extensive datasets^[24]^. This study aims to develop and evaluate an ML algorithm to predict the success of a second-look M-TESE, potentially improving clinical decision-making and patient outcomes.

## Methods

The dataset consists of medical records from 47 patients who have undergone micro-testicular sperm extraction (microTESE). Tables 1 and 2 provides an overview of the variables in the dataset along with their respective data types. A multivariate analysis was conducted using Matplotlib, a Python library for data visualization. This analysis allowed us to examine the relationships between the variables and the efficacy of a second microTESE. This work has been reported in line with the STROCSS criteria^[25]^.

**Table 1:** Overview of variables collected from medical records of 47 patients who underwent microTESE

Variable name	Data type	Description
Procedure side	Categorical	Side of the testes where the procedure was done
Date	Numerical	The date of the second surgery
Result	Categorical	The outcome of the second Micro TESE
Histopathology	Categorical	
Previous	Numerical	Date of the previous surgery
Preop-FSH	Numerical	The amount of FSH before the surgery in mIU/ml
Preop testosterone	Numerical	The amount of testosterone in the patient prior to the surgery
Testicular volume	Numerical	The volume of testicular fluid
Klinefelter’s syndrome	Categorical	Associated Klinefelter Syndrome
Cryptorchidism	Categorical	Associated Cryptorchidism
Cancer history	Categorical	If there is previous history of cancer to any body organ
Varicocele	Categorical	Associated varicocele
Idiopathic NOA	Categorical	
Age (y)	Numerical	The age of the patient

**Table 2:** Descriptive statistics of age and hormonal parameters in the study population

Variables	Mean ± SD	Class	Frequency
Age	39.68 ± 6.39		
Preop FSH	23.1 ± 21.3	High	30
		Normal	15
		Low	2
Preop testosterone	15.49 ± 8.04	High	1
		Normal	32
		Low	15

## Data preparation

The dataset consisted of eleven categorical variables. These variables were converted to integers using the Label Encoder. There were no missing values, and duplicate entries were removed. The data were split into training and testing sets in an 80:20 ratio due to the small sample size.

## Performance evaluation

Several supervised ML algorithms were employed in this study, including support vector machine (SVM), logistic regression, XGBoost regressor, and random forests. The input variables comprised independent attributes such as procedure side, histopathology, preoperative FSH, preoperative testosterone, testicular volume, Klinefelter’s syndrome, cryptorchidism, cancer history, varicocele, idiopathic Non Obstructive Azoospermia (NOA), age, FSH classification, testosterone classification, and interval. The outcome of the second-look microTESE was recorded as binary: 1 (positive) and 0 (negative). The ML models were implemented using the scikit-learn 0.18 module in Python.

The original dataset was randomly divided into 80% for the training set and 20% for the test set.

## Support vector model

SVM applies the concept of mapping vectors to a high-dimensional feature space to create a linear decision surface (hyperplane) that separates classes. This approach is used for binary classification and can effectively solve both linearly separable and non-separable problems (Wang *et al*, 2017). SVMs have demonstrated strong generalization and learning capabilities, making them a popular ML method.

SVMs modify the simple mathematical formula *y = wx′ + by = wx′ + b* to enable linear domain division. There are two types of SVMs: linear and nonlinear. A linear SVM is used when the dataset can be partitioned linearly, while a nonlinear SVM is used when it cannot. Nonlinear SVMs transform the data domain into a feature space, allowing for linear separation of the classes (Shafi & Shah, 2017). The goal of SVM is to find the best hyperplane that accurately divides the dataset. In linear cases, the dimension of the hyperplane is *n*, while in nonlinear cases, it is *n*−1.

Mathematically, it is represented as:

For linear cases: *y = wx′ + by = wx′ + b*

For nonlinear cases: *y = wϕ(x′) + yy = wϕ(x′) + y*

For this experiment, the learning rate for the models was tuned to 0.001, and GridSearchCV was used to select the best parameters for model training. This strategy allowed the predictive model to learn using the best features and parameters from the dataset, build individual weak models, and generate a stronger model by combining several weak models. The area under the curve was calculated using the sklearn.metric module in Python.

**Figure 1.**
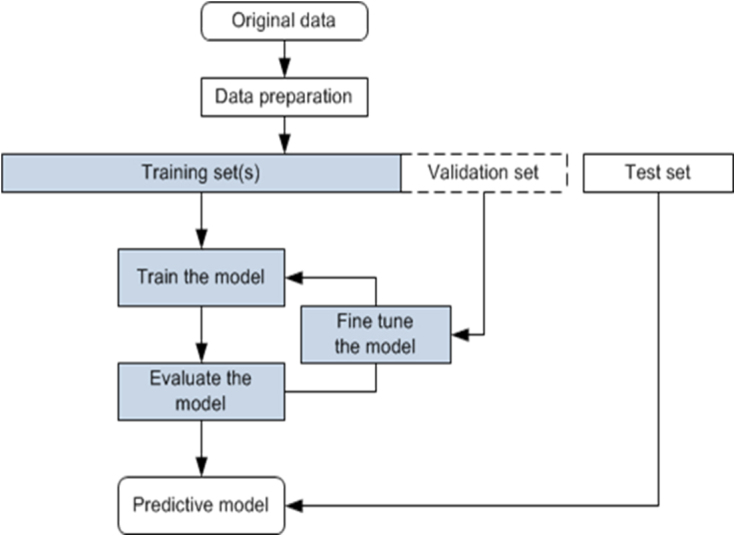
Relationship between the interval between previous and current surgery and microTESE outcome. Patients with shorter intervals tended to have negative results, while longer intervals correlated with positive outcomes, suggesting recovery time improves efficacy.

Figure 1 shows the relationship between the interval between the previous and current surgery and the result. It can be observed that patients with a shorter interval between surgical interventions tended to have negative results, indicating that repeated short-interval surgeries may be less effective. In contrast, patients with longer intervals tended to have more positive results, likely due to the time allowing for recovery or relapse reduction.

## Results

### Descriptive statistics

**Figure 2.**
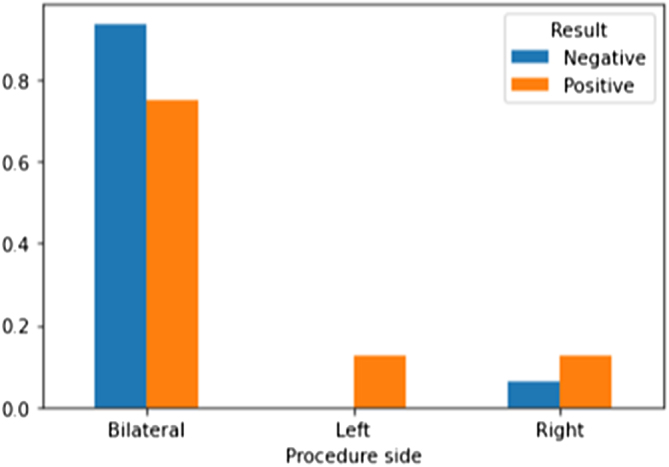
Correlation between procedure side (unilateral vs. bilateral) and microTESE outcome. Bilateral procedures were associated with higher success rates.

**Figure 3.**
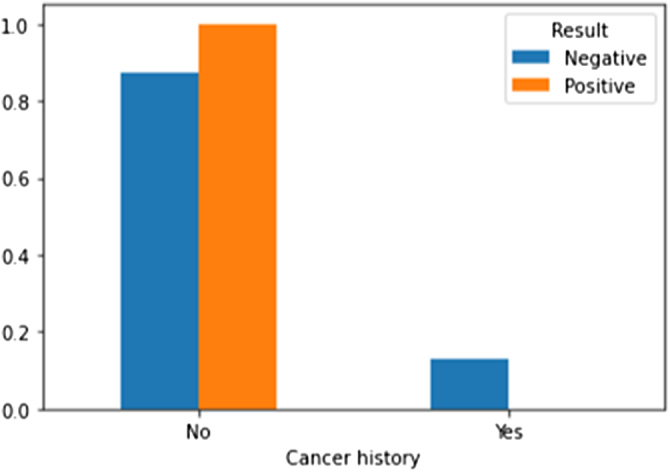
Association between prior cancer history and microTESE outcome. All patients with a cancer history had negative results, indicating a potential risk factor for failure.

**Figure 4.**
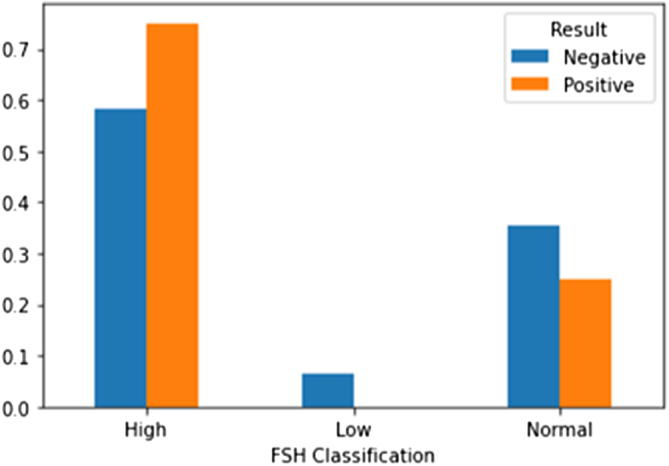
Stratification of patients by Follicle-stimulating hormone (FSH) levels. Positive outcomes were more frequent in patients with high FSH (>15.4 mIU/mL), while low FSH correlated with negative results.

A multivariate analysis was conducted using Matplotlib, a Python library for data visualization. This analysis enabled us to understand the relationships between the variables and the efficacy of a second microTESE. Figures 2–4 illustrate the multivariate analysis of selected comorbidities and the outcomes of the second microTESE.

Figure 2 shows that the majority of patients who had a positive result after the second surgery underwent the procedure bilaterally. This suggests that bilateral procedures may have a higher success rate compared to unilateral procedures.

Figure 2: Correlation between procedure side and micro-TESE.

Figure 3 shows that previous cancer history has a correlation with the outcomes of microTESE. All those who had a previous history of cancer had negative results after the test, indicating that a history of cancer might be a risk factor for failure in microTESE.

Figure 4 stratifies patients based on their FSH levels. It was established that the majority of those with a positive result had high FSH levels (greater than 15.4 mIU/mL), while those with low FSH levels did not have positive outcomes.

**Figure 5.**
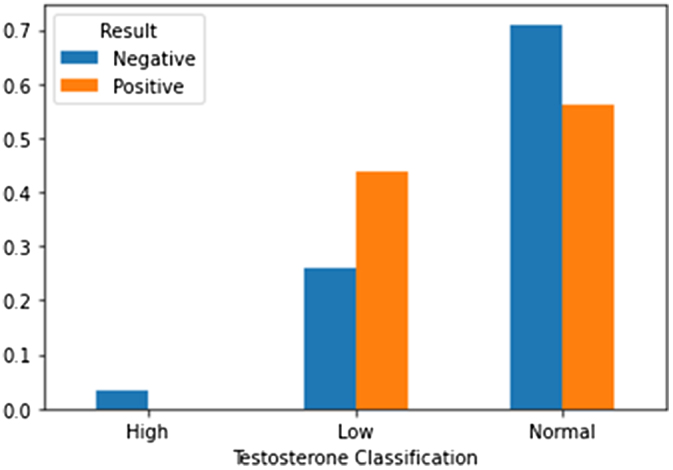
Testosterone level stratification and microTESE outcomes. Patients with low testosterone (<10 nmol/L) had higher positive result rates, whereas normal/high levels were linked to negative outcomes.

Figure 5 shows the stratification of patients based on testosterone levels. It was noted that the majority of patients with positive results for azoospermia had low testosterone levels (less than 10 nmol/L). Patients with normal or high testosterone levels were more likely to have negative results.

**Figure 6.**
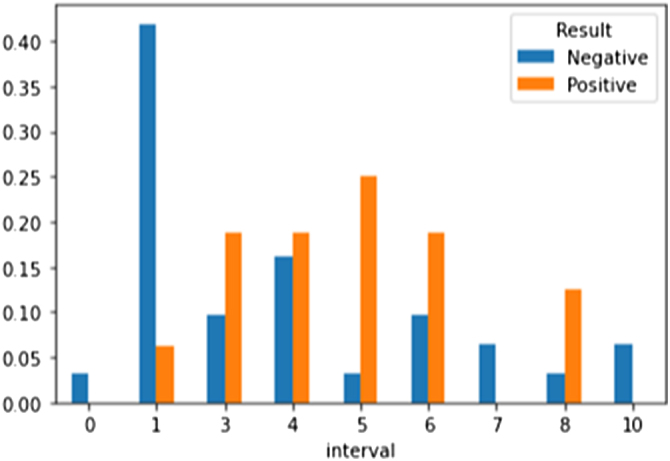
Reiteration of interval-surgery relationship (similar to Fig. 1). Shorter intervals corresponded to negative results, while longer intervals improved success, likely due to recovery time.

Figure 6 illustrates the relationship between the interval between the previous and current surgery and the results. It was observed that patients with shorter intervals between surgeries had negative results, indicating the inefficacy of repeated short-interval surgeries. In contrast, patients with longer intervals had more positive results, likely due to the time allowing for recovery or reduction in recurrence.

### Performance evaluation

**Figure 7.**
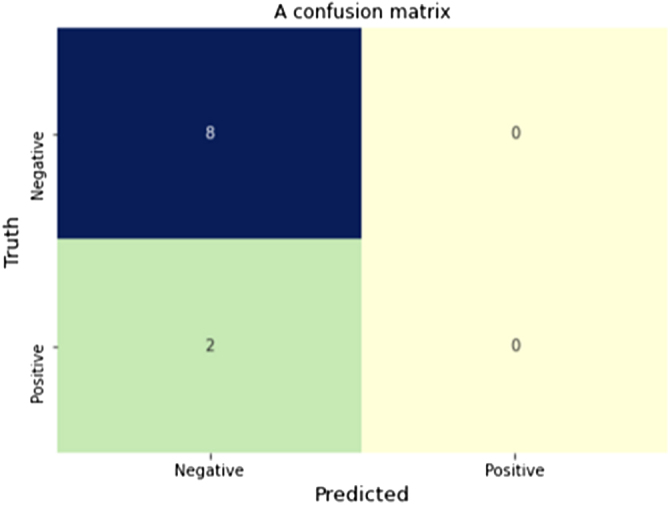
Confusion matrix of the support vector machine (SVM) model after hyperparameter tuning, demonstrating 80% accuracy in predicting microTESE outcomes.

The SVM used in this study achieved an accuracy of 80% after hyperparameter tuning to reduce overfitting. Figure 7 shows the confusion matrix of the SVM model. This confusion matrix indicates that the SVM model is fairly precise in its predictions.

**Figure 8.**
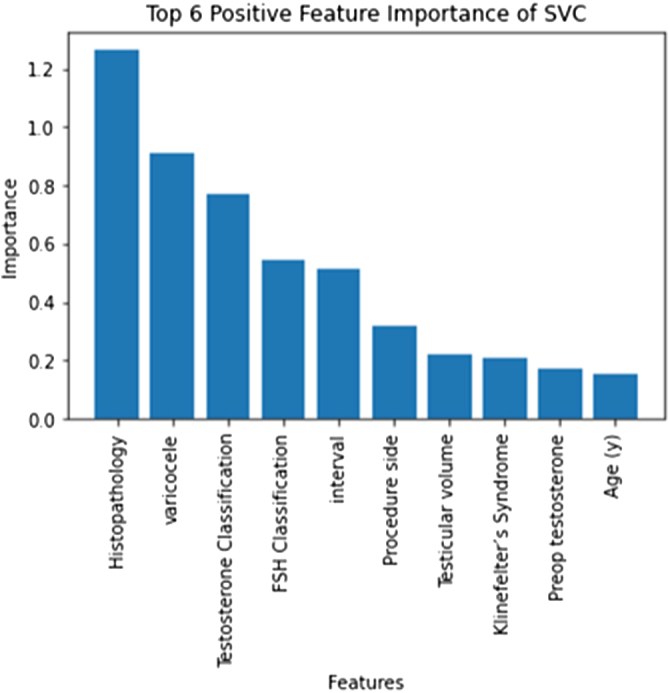
Feature importance ranking from the SVM model. Histopathology, varicocele, testosterone, FSH levels, and inter-surgery interval were strongly correlated with second microTESE success.

Figure 8 shows the feature importance for the SVM. It is noted that histopathology, varicocele, testosterone levels, FSH levels, and the interval between procedures are highly correlated with the probability of a successful second microTESE.

## Discussion

ML methods can be invaluable in predicting TESE outcomes because they analyze the relationships between various variables in the training set^[26]^. Predictive factors for the success of microTESE have been extensively studied, with several key factors consistently identified across research studies.

Preoperative testosterone serum levels after medical treatment have been shown to predict success at sperm retrieval by microTESE in men with Klinefelter syndrome^[9]^. Younger age, better semen parameters, favorable histological features, and lower FSH values have been associated with shorter time to sperm retrieval and successful outcomes^[12]^. Additionally, testicular volume and serum FSH levels have been identified as positive predictive factors for sperm recovery^[27]^. Cissen *et al*^[28]^ also identified age and FSH levels as significant predictors for successful TESE. The expression ratio of histone demethylase KDM3A to protamine-1 mRNA has been proposed as a reliable marker for successful TESE in men with both obstructive and non-obstructive azoospermia^[29]^. Conversely, high FSH levels and small testicular volume have been significantly associated with lower chances of successful sperm retrieval^[30]^. Testicular histopathology type influences the success of salvage microTESE, with hypospermatogenesis being a positive predictor and maturation arrest being a negative predictor^[31]^. However, inhibin B levels were not found to be a reliable predictor for TESE success^[32]^.

Our ML model accurately predicted the presence or absence of spermatozoa in patients with NOA. We found that patients who underwent bilateral microTESE had a higher likelihood of a positive result after a second M-TESE. Additionally, a previous history of cancer was correlated with a negative result after the procedure. Furthermore, FSH and testosterone levels were also identified as predictive factors. The performance evaluation of the SVM model demonstrated an accuracy of 80%, indicating its effectiveness in predicting the success of a second M-TESE based on factors such as histopathology, varicocele, testosterone levels, FSH levels, and the interval between surgeries. These findings are consistent with previous studies, further supporting their validity. The integration of the SVM model into clinical practice can significantly enhance decision-making processes for clinicians. For instance, a clinician could use the model to assess a patient with a history of non-obstructive azoospermia considering a second microTESE. By entering relevant factors such as age and hormonal profiles into the model, the clinician could receive a predictive score indicating the likelihood of sperm retrieval success, fostering informed discussions with patients about their options and managing expectations.

However, our study has several limitations that should be acknowledged. First, the retrospective nature of this study and the limited sample size introduce potential selection bias, which must be carefully considered when interpreting the results. Future studies should aim to validate our model prospectively and with a larger, more diverse population to ensure broader applicability and to mitigate selection bias. Second, the study relied on retrospective data, which may be subject to errors or missing information. Furthermore, although our study involves a limited sample size of 47 patients, which restricts the generalizability of the findings, this size aligns with similar studies in the existing literature that utilized comparable cohorts for initial model development ^[33,34]^. Previous studies have indicated that utilizing ML techniques on small datasets can yield valuable insights when reinforced with cross-validation and hyperparameter tuning. Despite these limitations, our study provides valuable insights into the predictive factors for the success of a second M-TESE procedure.

The findings suggest that bilateral microTESE increases the likelihood of success in a second M-TESE procedure. Additionally, a history of cancer correlates with negative outcomes, indicating this factor should be considered when assessing the potential success of a second M-TESE procedure^[35]^. Hormonal levels, particularly FSH and testosterone, play a crucial role in the outcome, underscoring their importance in preoperative evaluation. Based on these findings, the SVM model showed promising accuracy in predicting the success of a second M-TESE procedure.

## Conclusion

In conclusion, ML models, particularly the SVM, show promise in predicting the success of a second M-TESE procedure. By incorporating factors such as age, hormonal levels, testicular volume, and genetic evaluation, our model can help clinicians and patients make informed decisions, potentially improving outcomes and reducing unnecessary procedures. Further validation and refinement are needed to ensure the model’s accuracy and applicability across different populations.

## Data Availability

All data are mentioned in the manuscript.

## Research registration unique identifying number (UIN)

Research Registry, researchregistry10459, https://www.researchregistry.com/browse-theregistry#home/registrationdetails/668a9bc9f486120027b280f5/.

## References

[1] Jarow JP Espeland MA Lipshultz LI. Evaluation of the azoospermic patient. J Urol 1989;142:62–65. 2499695 10.1016/s0022-5347(17)38662-7

[2] Ramasamy R Schlegel PN. Microdissection testicular sperm extraction: effect of prior biopsy on success of sperm retrieval. J Urol 2007;177:1447–49. 17382751 10.1016/j.juro.2006.11.039

[3] SU LM Palermo GD GOLDSTEIN M. Testicular sperm extraction with intracytoplasmic sperm injection for nonobstructive azoospermia: testicular histology can predict success of sperm retrieval. J Urol 1999;161:112–16. 10037381

[4] Bernie AM Mata DA Ramasamy R. Comparison of microdissection testicular sperm extraction, conventional testicular sperm extraction, and testicular sperm aspiration for nonobstructive azoospermia: a systematic review and meta-analysis. Fertil Steril 2015;104:1099–103. 26263080 10.1016/j.fertnstert.2015.07.1136

[5] Abdel Raheem A Garaffa G Rushwan N. Testicular histopathology as a predictor of a positive sperm retrieval in men with non-obstructive azoospermia. BJU Int 2013;111:492–99. 22583840 10.1111/j.1464-410X.2012.11203.x

[6] Huyghe E Boitrelle F Methorst C. AFU and SALF recommendations for the evaluation of male infertility. Progres En Urol J Assoc Francaise Urol Soc Francaise Urol 2020;31:131–44. 10.1016/j.purol.2020.09.011 33309127

[7] Tsujimura A Miyagawa Y Takao T. Salvage microdissection testicular sperm extraction after failed conventional testicular sperm extraction in patients with nonobstructive azoospermia. J Urol 2006;175:1446–49. 16516017 10.1016/S0022-5347(05)00678-6

[8] Talas H Yaman O Aydos K. Outcome of repeated micro-surgical testicular sperm extraction in patients with non-obstructive azoospermia. Asian J Androl 2007;9:668–73. 17712484 10.1111/j.1745-7262.2007.00273.x

[9] Dabaja AA Schlegel PN. Microdissection testicular sperm extraction: an update. Asian J Androl 2013;15:35. 23241638 10.1038/aja.2012.141 PMC3739122

[10] Shiraishi K Ohmi C Shimabukuro T. Human chorionic gonadotrophin treatment prior to microdissection testicular sperm extraction in non-obstructive azoospermia. Hum Reprod 2012;27:331–39. 22128297 10.1093/humrep/der404

[11] Shiraishi K Ishikawa T Watanabe N. Salvage hormonal therapy after failed microdissection testicular sperm extraction: a multi-institutional prospective study. Int J Urol 2016;23:496–500. 26989893 10.1111/iju.13076

[12] Gnessi L Scarselli F Minasi MG. Testicular histopathology, semen analysis and FSH, predictive value of sperm retrieval: supportive counseling in case of reoperation after testicular sperm extraction (TESE). BMC Urol 2018;18:1–8. 29973189 10.1186/s12894-018-0379-7 PMC6032772

[13] Tsujimura A Matsumiya K Miyagawa Y. Prediction of successful outcome of microdissection testicular sperm extraction in men with idiopathic nonobstructive azoospermia. J Urol 2004;172:1944–47. 15540761 10.1097/01.ju.0000142885.20116.60

[14] Mitchell V Robin G Boitrelle F. Correlation between testicular sperm extraction outcomes and clinical, endocrine and testicular histology parameters in 120 azoospermic men with normal serum FSH levels. Int J Androl 2011;34:299–305. 20695924 10.1111/j.1365-2605.2010.01094.x

[15] Anniballo R Ubaldi F Cobellis L. Criteria predicting the absence of spermatozoa in the Sertoli cell-only syndrome can be used to improve success rates of sperm retrieval. Hum Reprod 2000;15:2269–77. 11056118 10.1093/humrep/15.11.2269

[16] Hopps C Mielnik A Goldstein M. Detection of sperm in men with Y chromosome microdeletions of the AZFa, AZFb and AZFc regions. Hum Reprod 2003;18:1660–65. 12871878 10.1093/humrep/deg348

[17] Foresta C Moro E Ferlin A. Y chromosome microdeletions and alterations of spermatogenesis. Endocr Rev 2001;22:226–39. 11294825 10.1210/edrv.22.2.0425

[18] Raman JD Schlegel PN. Testicular sperm extraction with intracytoplasmic sperm injection is successful for the treatment of nonobstructive azoospermia associated with cryptorchidism. J Urol 2003;170:1287–90. 14501743 10.1097/01.ju.0000080707.75753.ec

[19] Barbotin AL Dauvergne A Dumont A. Bilateral versus unilateral cryptorchidism in nonobstructive azoospermia: testicular sperm extraction outcomes. Asian J Androl 2019;21:445–51. 30880688 10.4103/aja.aja_2_19 PMC6732891

[20] Corona G Minhas S Giwercman A. Sperm recovery and ICSI outcomes in men with non-obstructive azoospermia: a systematic review and meta-analysis. Hum Reprod Update 2019;25:733–57. 31665451 10.1093/humupd/dmz028

[21] Qi L Liu YP Zhang NN. Predictors of testicular sperm retrieval in patients with non-obstructive azoospermia: a review. J Int Med Res 2021;49:03000605211002703. 33794677 10.1177/03000605211002703 PMC8020245

[22] Ramasamy R Lin K Gosden LV. Reprint of: high serum FSH levels in men with nonobstructive azoospermia does not affect success of microdissection testicular sperm extraction. Fertil Steril 2019;112:e67–70. 31623744 10.1016/j.fertnstert.2019.08.075

[23] Kavoussi PK West BT Chen SH. A comprehensive assessment of predictors of fertility outcomes in men with non-obstructive azoospermia undergoing microdissection testicular sperm extraction. Reprod Biol Endocrinol 2020;18:1–8. 32847601 10.1186/s12958-020-00646-4 PMC7448981

[24] Bachelot G Lévy R Bachelot A. Proof of concept and development of a couple-based machine learning model to stratify infertile patients with idiopathic infertility. Sci Rep 2021;11:24003. 34907216 10.1038/s41598-021-03165-3 PMC8671584

[25] Mathew G Agha R Albrecht J. STROCSS 2021: strengthening the reporting of cohort, cross-sectional and case-control studies in surgery. Int J Surg 2021;96:106165. 34774726 10.1016/j.ijsu.2021.106165

[26] Blank C Wildeboer RR DeCroo I. Prediction of implantation after blastocyst transfer in in vitro fertilization: a machine-learning perspective. Fertil Steril 2019;111:318–26. 30611557 10.1016/j.fertnstert.2018.10.030

[27] Seo JT Ko WJ. Predictive factors of successful testicular sperm recovery in non-obstructive azoospermia patients. Int J Androl 2001;24:306–10. 11554989 10.1046/j.1365-2605.2001.00307.x

[28] Cissen M Meijerink AM D’Hauwers KW. Prediction model for obtaining spermatozoa with testicular sperm extraction in men with non-obstructive azoospermia. Hum Reprod Oxf Engl 2016;31:1934–41. 10.1093/humrep/dew147 27406950

[29] Javadirad S Hojati Z Ghaedi K. Expression ratio of histone demethylase KDM 3A to protamine-1 mRNA is predictive of successful testicular sperm extraction in men with obstructive and non-obstructive azoospermia. Andrology 2016;4:492–99. 27027467 10.1111/andr.12164

[30] Salehi P Derakhshan-Horeh M Nadeali Z. Factors influencing sperm retrieval following testicular sperm extraction in nonobstructive azoospermia patients. Clin Exp Reprod Med 2017;44:22. 28428940 10.5653/cerm.2017.44.1.22 PMC5395548

[31] Zhang F Dai M Yang X. Predictors of successful salvage microdissection testicular sperm extraction (mTESE) after failed initial TESE in patients with non-obstructive azoospermia: a systematic review and meta-analysis. Andrology 2024;12:30–44. 37172416 10.1111/andr.13448

[32] von Eckardstein S Simoni M Bergmann M. Serum inhibin B in combination with serum follicle-stimulating hormone (FSH) is a more sensitive marker than serum FSH alone for impaired spermatogenesis in men, but cannot predict the presence of sperm in testicular tissue samples. J Clin Endocrinol Metab 1999;84:2496–501. 10404826 10.1210/jcem.84.7.5855

[33] Ramasamy R Reifsnyder JE Husseini J. Localization of sperm during microdissection testicular sperm extraction in men with nonobstructive azoospermia. J Urol 2013;189:643–46. 23260549 10.1016/j.juro.2012.09.031

[34] Kalsi JS Shah P Thum Y. Salvage micro-dissection testicular sperm extraction; outcome in men with non-obstructive azoospermia with previous failed sperm retrievals. BJU Int 2015;116:460–65. 25220441 10.1111/bju.12932

[35] Devroey P Liu J Nagy Z. Pregnancies after testicular sperm extraction and intracytoplasmic sperm injection in non-obstructive azoospermia. Hum Reprod 1995;10:1457–60. 7593514 10.1093/humrep/10.6.1457

